# Long-term survival of cancer patients compared to heart failure and stroke: A systematic review

**DOI:** 10.1186/1471-2407-10-105

**Published:** 2010-03-22

**Authors:** Vasileios Askoxylakis, Christian Thieke, Sven T Pleger, Patrick Most, Judith Tanner, Katja Lindel, Hugo A Katus, Jürgen Debus, Marc Bischof

**Affiliations:** 1 Department of Radiooncology and Radiation Therapy, University of Heidelberg, Im Neuenheimer Feld 400, 69120, Heidelberg, Germany; 2 Department of Radiation Therapy, German Cancer Research Center, Im Neuenheimer Feld 280, 69120, Heidelberg, Germany; 3 Department of Internal Medicine III, University of Heidelberg, Im Neuenheimer Feld 410, 69120, Heidelberg, Germany

## Abstract

**Background:**

Cancer, heart failure and stroke are among the most common causes of death worldwide. Investigation of the prognostic impact of each disease is important, especially for a better understanding of competing risks. Aim of this study is to provide an overview of long term survival of cancer, heart failure and stroke patients based on the results of large population- and hospital-based studies.

**Methods:**

Records for our study were identified by searches of Medline via Pubmed. We focused on observed and relative age- and sex-adjusted 5-year survival rates for cancer in general and for the four most common malignancies in developed countries, i.e. lung, breast, prostate and colorectal cancer, as well as for heart failure and stroke.

**Results:**

Twenty studies were identified and included for analysis. Five-year observed survival was about 43% for all cancer entities, 40-68% for stroke and 26-52% for heart failure. Five-year age and sex adjusted relative survival was 50-57% for all cancer entities, about 50% for stroke and about 62% for heart failure. In regard to the four most common malignancies in developed countries 5-year relative survival was 12-18% for lung cancer, 73-89% for breast cancer, 50-99% for prostate cancer and about 43-63% for colorectal cancer. Trend analysis revealed a survival improvement over the last decades.

**Conclusions:**

The results indicate that long term survival and prognosis of cancer is not necessarily worse than that of heart failure and stroke. However, a comparison of the prognostic impact of the different diseases is limited, corroborating the necessity for further systematic investigation of competing risks.

## Background

The facts that multiple diseases are present in many patients and that this trend is expected to increase in the future due to population ageing reveal the necessity for a better understanding of competing risks. Among the diseases with high mortality cancer, heart failure and stroke represent major global healthcare problems.

Cancer is the second most common cause of death after cardiovascular diseases. According to the World Health Organization (WHO), more than 10 million people are diagnosed with cancer yearly. The disease is responsible for 6 million deaths per year accounting for up to 12% of all cases. Fifty six percent of newly diagnosed cancer patients are >65 years, while about 70% of cancer deaths are in this age group. The median age of cancer patients at death for both sexes ranges from 71 to 77 years [1]. The four most common malignancies in developed countries are lung, breast, prostate, and colorectal cancer. These account for nearly half of all incident cases and cancer deaths of the total European cancer burden [2]. The most common entity overall and the leading cause of cancer related mortality is lung cancer. Worldwide about 1.35 million new cases and about 1.18 million deaths are estimated annually [3]. Among women the most common entity in developed countries is breast cancer. The disease is diagnosed in about 1.2 million patients and accounts for about 500,000 deaths yearly in the world [4]. Prostate cancer represents the most common cancer in men in developed countries with estimations for 2007 revealing about 782,600 new cases and 254,000 deaths [5,6]. Finally colorectal cancer is the third most common malignancy worldwide with the WHO estimating about 945,000 new cases and 492,000 deaths annually [7].

Heart failure is a growing cause of morbidity and mortality among cardiovascular diseases, with increasing prevalence in recent years [8]. Despite significant improvements, the disease continues to represent an enormous clinical challenge. Currently, about 5 million patients are suffering in the USA from heart failure, while more than 550,000 are diagnosed yearly [9]. Incidence of the disease approaches 10 per 1000 population after age 65 and mortality was about 300,000 cases in 2006 in the USA [10]. About 80% of patients with new diagnosed heart failure are >65 years old and 50% are >75 years old. The disease is currently one of the most frequent causes of hospitalization, with the annual number in the USA estimated to be over 1 million [11].

The third leading cause of death in developed countries represents cerebrovascular disease. In 2002, stroke was the cause of 5.5 million deaths worldwide, accounting for about 10% of total deaths. The prevalence of stroke in the USA is over 700,000 cases per year [12]. About 75% of all first stroke events occur after the age of 65 years [13]. The disease is associated with high mortality in the acute phase [14]. The annual risk of death after stroke is about 10% [15,16], while the annual recurrence risk is 5% per year, similar to that seen in patients with coronary events [17].

Aim of the present article is to provide an overview of long-term survival of cancer, heart failure and stroke patients, based on the results of large locoregional and international studies. The choice of those diseases in our analysis is based on the epidemiological data concerning incidence, prevalence and mortality that underline the high impact of cancer, heart failure and stroke in public health and show that the diseases tend to affect patients of similar age.

## Methods

### Search strategy

We identified published studies investigating long-term survival of cancer, heart failure and stroke patients after diagnosis in different developed countries using electronic search strategies. The reference lists of identified articles were also screened and experts in the field were contacted. The comprehensive literature search was performed between February and August 2009.

Records for our study were identified by searches of MEDLINE via PubMed. Key-words used were "long-term survival", "5-year relative survival", "breast cancer", "prostate cancer", "colorectal cancer", "lung cancer", "heart failure", and "stroke". The key-words were combined using the Boolean operator "and" between survival and diagnosis type keywords.

### Study selection criteria

We included large, population-based and hospital-based studies from developed countries that reported observed five-year survival and/or age- and sex adjusted five-year relative survival rates of the four most frequent cancer entities in developed countries, i.e. lung, breast, prostate and colorectal cancer, heart failure and stroke after first diagnosis. Included were studies published between 2003 and 2009. All languages and types of publications were considered eligible.

We excluded review articles and studies that investigated long-term survival restricted to a specific stage of the disease, or patients who received a specific treatment. Excluded were also studies investigating a specific patient collective, i.e. patients living under specific socioeconomic circumstances as well as studies investigating collectives of less than 200 patients. Furthermore, studies with short follow-up period (shorter than 5 years) and studies with unclear characteristics and methodology, i.e. studies which did not report the methods used for evaluation of long-term survival, such as follow-up of the collective or methodology of statistical analysis were excluded.

### Data extraction and assessement of methodological quality

One reviewer (VA) screened all titles and abstracts to determinate whether the research article fulfilled the inclusion criteria. Full reports from the selected articles were retrieved by two reviewers (VA and CT) using the same criteria as for the initial selection. Data extracted included demographic characteristics, study period, identifying information and focus of the study. Primary endpoint was 5-year observed and/or 5-year age- and sex adjusted relative survival.

Methodological quality of the selected studies was assessed by two reviewers (VA and CT). Primary item used to assess study quality was the methodology applied for the determination of long-term survival. The applied methodology was deemed appropriate when follow-up was adequately completed for a time period of at least 5 years after first ever diagnosis and when survival calculation was based on period analysis methodology. Relative survival estimates were calculated as the ratio of observed to expected survival, based on calendar-year, sex and age specific life tables.

## Results

### Identification of studies and study characteristics

**Figure 1:**
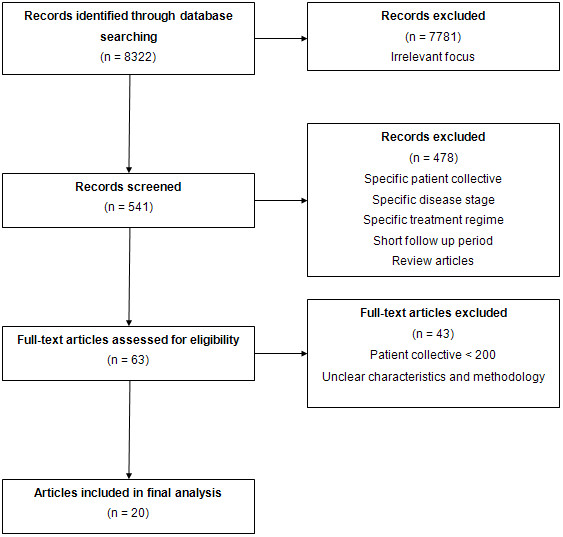
**Flow diagram of the study selection process**.

A flow diagram of the study selection process is presented in Figure 1. At the end of the selection process 20 unique citations were identified to fulfill inclusion criteria. Those citations were identified by the electronic search strategy and are published in peer-reviewed journals in the years 2003-2009. Five-year observed and/or five-year relative survival was investigated after diagnosis or first hospitalization for each disease. An overview of the characteristics and the results of the studies included in the analysis is presented in Additional file 1. Special aspects of the included studies are presented in the next paragraphs.

### Long-term survival in cancer patients

Long-term survival for cancer patients in Europe has been extensively investigated within the EUROCARE series [18-20]. Recently, the results of the EUROCARE-4 study, analyzing long-term survival of about 3 million adult cancer patients diagnosed in 1995-1999, were published [21]. Data for the population were obtained from 82 cancer registries from 23 European countries. Women had a better outcome, with 5-year observed and relative survival of 50 and 58.2%, respectively (36.8 and 45.9% for men). Among all patients aged 65-74 years, the observed 5-year survival was 41.3%; the value for patients >75 years was 24.1%. Five-year relative survival was 48.1% for the age group 65-74 years and 40.1% for patients >75 years.

Gondos et al analyzed data from the Saarland Cancer Registry in Germany. Five-year relative survival was 53.9% for patients aged 65-74 years and 47.1% for patients >75 years. The same study revealed an increased 5-year relative survival over time from 42.2% in 1979-1983 to 56.7% in 1999-2003 [22].

### Lung cancer

In the EUROCARE-4 study, 5-year observed and relative survivals were 9.5 and 11.2%, respectively for patients aged 65-74 years and 4.3 and 7.0% respectively for patients >75 years. A comparison with data from the EUROCARE-3 study (1990-1994) showed that survival has remained essentially unchanged [21].

In the study by Gondos et al [22] 5-year relative survival was 16.6 and 17% for the age groups 65-74 and >75 years, respectively. Compared to the period 1979-1983, a modest improvement in survival was identified.

Another study from the same group compared age-adjusted 5-year relative survival for 23 common forms of cancer between patients in Germany and the USA. Comparing the results of patients with lung cancer treated in Germany and patients treated in the USA from 2000 to 2002, similar results were found [23]. Grivaux et al presented the results of an epidemiological study including all new cases of histologically confirmed lung cancer managed in general hospitals in France. The study revealed 5-year observed survival of 10.4%. The median survival of the deceased patients was 7 months [24].

### Breast cancer

In the EUROCARE-4 study, 5-year observed and relative survival in patients aged 65-74 years were 72.4% and 80%, respectively. Even in older patients (>75 years) observed and relative 5-year survival rates were high (46.7% and 72%, respectively). The study revealed between-country survival differences with the highest age standardized 5-year relative survival in northern Europe countries (approximately 82%). Compared to the EUROCARE-3 study, an increasing survival trend was identified. Particularly, the mean European age- and area-standardized 5-year relative survival was 76% for the period 1990-1994 and 79% for the period 1995-1999 [21].

One of the first studies to provide a global comparison of cancer survival was the CONCORD study. The CONCORD study analyzed data from 101 population-based cancer registries from 31 countries. Within this study, age-standardized 5-year relative survival varied between different regions. The highest value was 83.7% for North America. Within the USA, 5-year relative survival ranged from 77.4% to 89.3%, while in Europe values varied from 57.9% to 82%. Among the 17 USA populations included in the study, survival was lower in blacks than in whites (70.9 vs 84.7%, respectively) [25].

Within the Michigan Cancer Surveillance Program, Meliker et al showed higher survival rates for the white population [26]. The racial disparity is confirmed by the results of other studies, revealing average 5-year survival rates of 78% for black and about 90% for white women with breast cancer in the USA [27].

### Prostate cancer

In the EUROCARE-4 study, 5-year observed and relative survival for patients aged 65-74 years were 66.9% and 81.1% and for patients aged 75-84 years 44.2% and 70.9%, respectively. Five-year age-standardized relative survival varied between European countries from 48% to about 87%. A trend towards improved 5-year survival was identified comparing age-standardized values of 65% in the EUROCARE-3 and 76% in the EUROCARE-4 study [21].

The CONCORD study revealed major regional differences in 5-year relative survival, especially in Europe. A race-specific analysis in the United States showed a higher survival rate for white (92.4%) than for black patients (85.8%) [25].

Gondos et al showed 5-year relative survival rates of 90.5% and 80.3% for patients aged 65-74 and >75 years, respectively. Compared to the period 1979-1983, improved survival rates were demonstrated [22].

The same group compared the results of prostate cancer patients in Germany and the USA. Age adjusted 5-year relative survival was 86.8% in Germany and 99.7% in the USA. According to the authors, this result could be explained by differences in screening intensity between the two countries [23].

Survival improvement over time was shown in a retrospective population-based study of prostate cancer cases reported to the Singapore Cancer Registry from 1968 to 2002. This study, performed by Chia et al, revealed a 7.9% increase in 5-year relative survival for 1998-2002 compared to 1978-1982 [28].

### Colorectal cancer

Within the EUROCARE-4 study, observed and relative 5-year survival was 46.6% and 54.3% for patients aged 65-74 years; for older patients (>75 years) the respective values were 28.5% and 47.6% [21].

In the study by Gondos et al [22] 5-year relative survival for all ages was 60% for colon cancer and 59.2% for rectal cancer, showing an increase of 16.3% and 19.5%, respectively, compared to previous time periods. In the second study by this group [23], patients in USA were found to have a moderately higher relative survival in all age groups compared to patients from Germany.

Survival analysis of patients with colorectal cancer in Sweden was performed by Birgisson et al, demonstrating a trend to improved relative survival rates for both colon and rectal cancer [29].

The CONCORD study revealed variability in colorectal cancer survival between different countries. The same study revealed racial disparities, with black populations having lower survival rates than white populations [25].

### Long-term survival for heart failure

Jhund et al investigated the long-term trends after first hospitalization for heart failure. The 5-year case-fatality rate for the entire patient population was 74%. Investigation of the trend over time revealed an outcome improvement. Five-year age-adjusted case fatality decreased from 73.7% to 65.8% in men and from 69.5% to 63.6% in women between 1986 and 1999 [30].

In a study by Blackledge et al, more than 60% of the patients were older than 75 years. Five-year all cause and cardiovascular survival for all patients were 27% and 41.8%, respectively. For the subpopulation of patients with heart failure as a primary diagnosis, 5-year all cause and cardiovascular survival were 24.4% and 37.9%, respectively. After six years of follow up, case fatality was 75-80% with worse outcome for patients with heart failure as a primary diagnosis [31].

Mahjoub et al investigated the long-term prognosis of patients with first heart failure episode in the Somme Department (France) during 2000. Five-year observed survival rates were 19% for octogenarians and 52% for younger patients, while 5-year relative survival for octogenarians and younger patients were 40 and 62%, respectively [32].

A study from the same region by Tribouilloy et al showed that 5-year survival for heart failure patients with preserved ejection fraction was equivalent to that of patients with reduced ejection fraction (43 vs. 46%, respectively) [33].

Similar results for patients with preserved and reduced ejection fraction were demonstrated by Owan et al; observed 5-year survival rates were 35 and 32%, respectively [34]. Thus, within two decades, improved pharmacological treatment and device technology increased survival of heart failure patients by 1 year, but 65% of all heart failure patients still died within 5 years.

### Long-term survival for stroke

Bravata et al investigated long term mortality in a cohort of patients aged >65 years who had been discharged with a primary diagnosis of acute ischemic stroke, transient ischemic attack, or carotid stenosis from Connecticut acute care hospitals in 1995. Annual mortality rate was 20.2% for the first year and decreased to about 12% for years 2-5. The authors showed that mortality rates depended on cerebrovascular diagnosis. Particularly, 5-year survival rate was 61.7% for carotid stenosis, 50.4% for TIA, and 40% for acute ischemic stroke [35].

Kim et al investigated long-term survival after first stroke in a nationally representative inpatient sample in Korea. Six-year survival rate was 65%. The most common causes of death were stroke and cardiovascular diseases, which were found to be responsible for 65.9% of deaths at 6-year follow-up [36].

Subanalysis of the results of a study performed by Reggiani et al revealed age and affected brain region as important prognostic factors. Five-year survival was 66.1% for patients aged 65-74 years and 49.4% for patients aged 75-84 years [37].

Vernino et al investigated mortality in patients after first cerebral infarction between 1985 and 1989. Most frequent causes of death were cardiovascular events (22%), respiratory infection (21%), and initial stroke complications (14%), while cancer accounted for 7.5% of deaths [38].

A retrospective study on patients with first ever stroke from 1996 to 1998 carried out by Cheung et al revealed 5-year mortality rates of 56.1% for patients with intracerebral haemorrhage and 33% for patients with ischemic stroke [39].

A population-based study investigating long-term survival after first ever stroke in Australia showed a 10-year cumulative risk of death of 79%. For the 10 years of follow-up, all patients had an about 3-times-greater risk of dying compared with individuals of the same age and sex in the general population [40]. A more recent study from the same group comparing 5-year survival and risk of recurrent stroke for the time periods 1989-90 and 1995-96 revealed no statistically significant survival improvement but a trend towards a smaller cumulative risk of recurrent stroke event [41].

## Discussion

Among diseases responsible for the majority of deaths worldwide, cancer, heart failure and stroke possess leading positions. Aim of this work is to provide an overview of long term survival of those diseases as described in large population- or hospital based studies from different developed countries. We chose cancer, heart failure and stroke for our analysis mainly because of their epidemiological characteristics. Incidence and mortality of the diseases demonstrate that they represent major global healthcare problems tending to affect patients of similar age. Furthermore, our clinical empiricism shows that in some cases the prognostic impact of each disease is unclear and that cancer is often considered to be the disease with the worst prognosis.

Most identified studies investigated 5-year observed survival for the disease of interest. In one of the largest population based studies, observed 5-year survival after diagnosis was about 43% for all cancer diseases [21]. Observed 5-year survival for heart failure varied between 26% and 52% and observed survival after a stroke event varied between 40% and 68%. However, comparison of observed survival has major limitations. The observed mortality rate within the cohort of interest does not represent the mortality rate associated only with the disease of interest, but is equal to the all-cause mortality rate in the reference population plus the excess mortality rate associated with the disease. Comparisons based on observed survival rates between different studies are not reliable, since they are affected by demographic differences between the different populations. In order to eliminate such effects calculation of relative survival rates is more appropriate. Relative survival rate is defined as the ratio of the observed survival rate in a group of patients to the survival rate expected in a group of people in the general population, who are similar to the patients with respect to all of the possible factors affecting survival at the beginning of the period, except for the disease under study [42]. A relative survival rate of 1 indicates that the mortality of the study group does not differ from that expected in the general population and that the mortality attributable to the disease is zero. For the calculation of relative survival rates the expected survival is usually estimated from nationwide population life tables, stratified by age, sex and calendar time. The major advantage of relative survival is that the information on cause of death is not required [43]. The analysis of the cancer related studies identified in our investigation demonstrated 5-year relative survival rates of 50% to 57% for all cancer entities. Differencies in 5-year relative survival was demonstrated for the different cancer entities. In particular, breast and prostate cancer showed 5-year relative survival rates of 73% to 89% and 50% to 99%, respectively, while lung cancer showed considerably lower survival rates (12% to about 18%). This result highlights the need to educate the patients and the general population that "cancer" is not one disease, but an umbrella term for a number of malignancies characterized by tissue infiltration and metastatic dissemination but manifold symptomatology, varying response to treatment strategies, and different long-term prognosis.

Five-year relative survival rates for heart failure and stroke in the studies identified in our search was about 62% and 50% respectively. A comparison to the relative survival rates of the different cancer entities indicates that cancer might not necessarily have a worse prognosis. However, this conclusion is strongly limited by various factors. The most important limiting parameter is the lack of appropriate data in regard to relative survival ratios for heart failure and stroke. Our search identified only two studies for each disease, which is not enough for safe conclusions. Furthermore, the use of relative survival rates underlies the assumption that patients are subject to two independent forces of mortality, i.e. that attributable to the disease and that in the general population. However, the disease of interest is often included in the reference population, resulting in bias of the estimation. Cancer and stroke are more closely linked to a hospital admission, while the development of screening techniques, such as PSA-screening for prostate cancer and mammography for breast cancer, has led to an earlier diagnosis of the diseases and therefore a better identification of patients and discrimination from the reference population. In case of heart failure, however, many patients are first diagnosed when symptoms become severe enough to require hospitalization. The patients that are not recognized are possibly included in the reference population, resulting in bias of the estimated relative survival rates.

Past studies have directly compared heart failure vs. cancer-related survival within the same population, showing results that are similar to the trend identified in our comparison. A study performed by Stewart et al investigated all patients with a first admission to any Scottish hospital in 1991 for heart failure and the four most common types of cancer, revealing that with the notable exception of lung cancer, heart failure was associated with a worse survival rate compared to other common cancer entities [44].

Although comparison and analysis of long term survival of different diseases is limited, it still can be an important tool for a better understanding of competing risks. The competing risks concept describes the analysis of how mortality trends of one disease might influence the mortality trends of another disease. To analyse this different models are available. The model of Chiang eliminates a specific cause in order to estimate the effect on mortality from other causes [45], while the model of Rothenberg is based on the assumption that mortality from competing causes remains constant [46]. The influence of cerebrovascular and cardiovascular disease mortality trends on cancer mortality trends has been analysed in various studies. Llorca et al analysed the interrelation between cerebrovascular disease, ischemic heart disease and cancer mortalities in Spanish women in 1981 and 1994 using both models and showed that although cerebrovascular and ischemic heart disease mortality have decreased in all age groups during the investigation period, this had not a significant impact on cancer mortality [47]. However, investigation of competing risks usually assumes independence between the different causes of death. Different causes of death are considered to be independent when they do not share common risk factors. A prominent example of a common risk factor between cardiovascular, cerebrovascular disease and various cancer entities is tobacco smoking. The error that is produced by the assumption of independence between those diseases is therefore influenced on the smoking prevalence of the investigated population and on the level of mortality from tobacco-related cancers [47]. The results of our systematic review cannot provide safe conlusions on competing risks, since they compare long term survival of different populations and do not provide information on risk factors that are important for analysis. Still, the competing risks problem shows the necessity for studies investigating outcome of different diseases, especially considering the fact that the number of multimorbid patients is expected to increase in the future mainly due to population ageing.

In respect to survival trends, the studies analysed here reveal results that are moderately encouraging. Comparison of long-term survival shows that the prognosis of cancer and heart failure has modestly improved over the last decades. However, a better understanding of the underlying molecular mechanisms of the diseases and their pathophysiology, leading to the development of new diagnostic and screening methods and to innovative therapeutic strategies, is still needed.

The comparison made here has some limitations. One is the lack of appropriate data in regard to relative survival rates of heart failure and stroke. Furthermore, most population-based studies use administrative data from registries and therefore miss detailed clinical information and stage-specific analysis. Retrospective studies with observational design are limited by inaccuracies in the diagnosis and coding of the diseases. The results of the analysis may be influenced by incomplete information concerning prior diagnosis, stage, and severity of the disease and variations in diagnostic accuracy and therapeutic approaches. Competing health risks of patients in clinical studies are not always fully analyzed because the final cause of death is not precisely reported.

## Conclusions

The analysis presented in this paper provides indications that cancer does not necessarily have a poorer prognosis compared to other common causes of death such as heart failure or stroke. Considering the fact that multiple diseases are present in many patients and that this trend is expected to increase in the future due to population ageing, there is an emerged need for a systematic investigation and statistical analysis of long term survival of different diseases within a population, coordinated by interdisciplinary teams. In this way, the role of competing risks will be better evaluated, which is important in order to better understand individual prognostic factors in multimorbid patients, prioritize the treatment of the prognostically leading disease and optimize therapeutic outcome.

## Competing interests

The authors declare that they have no competing interests.

## Authors' contributions

VA, CT and MB made substantial contribution to the conception and design of the study, data analysis, and interpretation, drafted the manuscript, and gave approval of the final version. STP, PM, JT, KL, HAK, and JD were involved in critically revising the manuscript for important intellectual content and gave approval of the final version.

## Pre-publication history

The pre-publication history for this paper can be accessed here:

http://www.biomedcentral.com/1471-2407/10/105/prepub

## Supplementary Material

Additional file 1 **Characteristics and results of the studies investigating 5-year survival of cancer, heart failure and stroke.** N/A: Not applicable, m: men, w: women. Click here for file
