# Supplementary material for: Long-term survival of cancer patients compared to heart failure and stroke: A systematic review
Source: BMC Cancer. 2010 Mar 22;10:105. doi: 10.1186/1471-2407-10-105 (PMC2851688; doi:10.1186/1471-2407-10-105)
Supplement: Additional file 1 — Characteristics and results of the studies investigating 5-year survival of cancer, heart failure and stroke. N/A: Not applicable, m: men, w: women. [file 1471-2407-10-105-S1.DOC]

| **Disease** | **Study (Reference)** | **Period** | **Region** | **Case-Number** | **Age (years)** | **5-year observed survival** | **5-year relative survival**  **(95% CI)** |
| --- | --- | --- | --- | --- | --- | --- | --- |
| **All cancer entities** | EUROCARE-4^21^ | 1995-1999 | Europe | 2,572,428 | ≥15 | 43.1% | 50.3% (50.2-50.5) |
|  | Gondos^22^ | 1999-2003 | Germany | 25,332 | ≥15 | N/A | 56.7% |
| **Lung** | EUROCARE-4^21^ | 1995-1999 | Europe | 338,755 | ≥15 | 10.1% | 12.0% (11.8-12.2) |
|  | Grivaux^24^ |  | France | 5,447 | All | 10.4% | N/A |
|  | Gondos^22^ | 1999-2003 | Germany | 3,040 | ≥15 | N/A | 17.5% |
|  | Gondos^23^ | 2000-2002 | USA | 36,869 | ≥15 | N/A | 15.5% |
| **Breast** | EUROCARE-4^21^ | 1995-1999 | Europe | 406,587 | ≥15 | 73.2% | 79.4% (79.1-79.7) |
|  | CONCORD^25^ | 1990-1994 | North America | 369,171 | 15-99 | N/A | 83.7% (83.5-83.9) |
|  |  |  | Europe | 303,830 | 15-99 | N/A | 73.1% (72.9-73.4) |
|  |  |  | Japan | 7,179 | 15-99 | N/A | 81.6% (79.7-83.5) |
|  |  |  | Australia | 41,090 | 15-99 | N/A | 80.7% (80.1-81.3) |
|  | Meliker^26^ | 1985-2002 | Michigan/USA | 124,218 | N/A | 78.0% | N/A |
|  | Gondos^22^ | 1999-2003 | Germany | 3,688 | ≥15 | N/A | 80.8% |
|  | Gondos^23^ | 2000-2002 | USA | 47,811 | ≥15 | N/A | 89.4% |
| **Prostate** | EUROCARE-4^21^ | 1995-1999 | Europe | 273,136 | ≥15 | 58.0% | 76.4% (75.9-76.9) |
|  | CONCORD^25^ | 1990-1994 | North America | 402,880 | 15-99 | N/A | 91.1% (90.0-91.3) |
|  |  |  | Europe | 161,771 | 15-99 | N/A | 57.1% (56.7-57.6) |
|  |  |  | Japan | 1,691 | 15-99 | N/A | 50.4% (46.3-54.9) |
|  |  |  | Australia | 42,890 | 15-99 | N/A | 77.4% (76.6-78.2) |
|  | Meliker^26^ | 1985-2002 | Michigan/USA | 120,615 | N/A | 75.0% | N/A |
|  | Gondos^22^ | 1999-2003 | Germany | 3,192 | ≥15 | N/A | 87.5% |
|  | Gondos^23^ | 2000-2002 | USA | 53,149 | ≥15 | N/A | 99.7% |
|  | Chia^28^ | 1998-2002 | Singapore | 1,351 | >20 | N/A | 60.9% (56.6-65.1) |
| **Colon** | EUROCARE-4^21^ | 1995-1999 | Europe | 228,221 | ≥15 | 42.8% | 54.5% (54.1-54.9) |
|  | CONCORD^25^ | 1990-1994 | North America | 213,667 | 15-99 | N/A | 59.5% m (59.1-59.9)  59.9% w (59.5-60.3) |
|  |  |  | Europe | 170,130 | 15-99 | N/A | 46.8% m (46.3-47.2)  48.4% w (48.0-48.8) |
|  |  |  | Japan | 10,057 | 15-99 | N/A | 63.0% m (61.3-64.8)  57.1% w (55.5-58.8) |
|  |  |  | Australia | 30,298 | 15-99 | N/A | 57.8% m (56.8-58.8)  57.7% w (56.7-58.6) |
|  | Birgisson^29^ | 1995-1999 | Sweden | 14,582 | All | 44.3% | 57.2% |
|  | Gondos^22^ | 1999-2003 | Germany | 4,301 | ≥15 | N/A | 60.0% |
| **Rectum** | EUROCARE-4^21^ | 1995-1999 | Europe | 138,232 | ≥15 | 43.2% | 53.2% (52.7-53.6) |
|  | CONCORD^25^ | 1990-1994 | North America | 83,415 | 15-99 | N/A | 56.4% m (55.8-56.9)  59.7% w (59.1-60.3) |
|  |  |  | Europe | 105,566 | 15-99 | N/A | 43.2% m (42.7-43.7)  47.4% w (46.9-48.0) |
|  |  |  | Japan | 5,758 | 15-99 | N/A | 58.2% m (55.9-60.5)  57.6% w (55.2-60.1) |
|  |  |  | Australia | 16,815 | 15-99 | N/A | 54.8% m (53.6-56.1)  59.2% w (57.8-60.6) |
|  | Birgisson^29^ | 1995-1999 | Sweden | 8,201 | All | 45.4% | 57.6% |
|  | Gondos^22^ | 1999-2003 | Germany | 4,301 | ≥15 | N/A | 59.2% |
| **Heart failure** | Jhund^30^ | 1986-2003 | Scotland | 116,556 | All | 26.0% | N/A |
|  | Blackledge^31^ | 1993-2001 | UK | 12,220 | ≥40 | 27.0% | N/A |
|  | Mahjoub^32^ | 2000 | France | 494 | <80 | 52.0% | 62.0% |
|  | Tribouilloy^33^ | 2000 | France | 662 | 25-100 | 44.0% | 61.0% |
|  | Owan^34^ | 1987-2001 | Minnesota/USA | 4,594 | All | 34.0% | N/A |
| **Stroke** | Bravata^35^ | 1995 | Conn./USA | 4,781 | ≥65 | 47.4% | N/A |
|  | Kim^36^ | 2000 | Korea | 4,299 | All | 67.7% | N/A |
|  | Reggiani^37^ | 1999 | Italy | 361 | All | 64.0% | N/A |
|  | Vernino^38^ | 1985-1989 | Minnesota/USA | 444 | All | 46.0% | N/A |
|  | Cheung^39^ | 1996-1998 | Hong Kong | 755 | 27-98 | 60.3% | N/A |
|  | Hardie^40^ | 1989-1990 | Australia | 251 | All | 40.6% | 48.2% |
|  | Hardie^41^ | 1995-1996 | Australia | 213 | All | 42.7% | 52.7% |
